# Fibrinogen-to-Albumin Ratio Predicts Contrast-Induced Nephropathy in Patients after Emergency Percutaneous Coronary Intervention

**DOI:** 10.1155/2019/8260583

**Published:** 2019-11-11

**Authors:** Zhebin You, Tailin Guo, Fan Lin, Chunjin Lin, Jiankang Chen, Xiaoming Li, Yan Chen, Kaiyang Lin

**Affiliations:** ^1^Department of Geriatric Medicine, Fujian Provincial Hospital, Fujian Key Laboratory of Geriatrics, Fujian Provincial Center for Geriatrics, Fujian Medical University, Fuzhou 350001, China; ^2^Department of Cardiology, Fujian Provincial Hospital, Fujian Medical University, Fujian Cardiovascular Institute, Fuzhou 350001, China

## Abstract

**Background:**

The aim of the present study was to investigate the association between fibrinogen-to-albumin ratio (FAR) with contrast-induced nephropathy (CIN) in patients undergoing emergency percutaneous coronary intervention (PCI).

**Methods:**

565 patients with emergency PCI were consecutively enrolled. The primary outcome was CIN defined as either a 25% increase in baseline serum creatinine levels or a 0.5 mg/dL (44 *μ*mol/L) increase in absolute serum creatinine levels within 72 h after the contrast medium exposure. Logistic regression analysis was applied to analyze whether FAR was an independent risk factor for CIN.

**Results:**

Overall, 29 (5.1%) patients developed CIN. Compared with the patients without CIN, the patients developing CIN had lower albumin (39.79 ± 3.95 vs. 37.14 ± 5.21, *P*=0.012) and higher fibrinogen levels (3.51 ± 0.94 vs. 4.14 ± 0.96, *P* < 0.001). In the multivariate logistic analysis, FAR was an independent predictor of CIN (OR = 3.97; 95% CI, 1.61–9.80; *P*=0.003) along with perihypotension, age >75 years, and LVEF <45%, and 0.106 was the optimal cutoff value of preprocedural FAR to predict CIN.

**Conclusion:**

Preprocedural levels of FAR were associated with CIN in patients after emergency PCI.

## 1. Introduction

Contrast-induced nephropathy (CIN) has become more frequent with the increased use of contrast media (CM), accounting for the third most common cause of hospital-acquired acute kidney injury (AKI) [1, 2]. CIN is associated with more in-hospital events, longer hospital stay, and increased risk of mortality, especially among patients undergoing emergency or primary percutaneous coronary intervention (PCI) [3–5]. The risk of CIN after emergency PCI is significantly increased than after elective PCI [6, 7]. Currently, there is no effective treatment for CIN. Therefore, identifying high-risk patients and early prophylactic measures are important for preventing CIN.

Several studies have confirmed that inflammation plays an important role in the initiation and procession of CIN [8, 9]. The systemic inflammation response-based indexes, such as neutrophil-to-lymphocyte ratio (NLR) and platelet-to-lymphocyte ratio (PLR), have been introduced as indicators of CIN in ST-elevated myocardial infarction (STEMI), non-ST-elevated myocardial infarction (NSTEMI), and coronary artery bypass graft (CABG) [10–14]. Fibrinogen-to-albumin ratio (FAR), combined with fibrinogen and albumin, was developed recently and has been demonstrated to be an effective and powerful prognostic indicator for several types of tumors [15–17]. Recently, Ertas et al. showed that FAR is an independent predictor in patients undergoing carotid angiography with 74.4% sensitivity and 60.8% specificity [18]. However, to our knowledge, no studies have explored the association of FAR with CIN and long-term outcomes in patients with emergency PCI.

The aim of this study was to investigate the role of FAR in predicting CIN and long-term outcome in patients undergoing emergency PCI.

## 2. Materials and Methods

### 2.1. Study Population

Between January 2012 and December 2015, consecutive patients who underwent an emergency PCI in Fujian Provincial hospital were enrolled in this study. Patients diagnosed with STEMI, or presented as high risk in those with non-ST-segment elevation acute coronary syndromes (i.e., those with refractory angina and hemodynamic instability), were selected for this study. The exclusion criteria included: (1) patients with pregnancy, lactation, and malignant tumor; (2) end-stage renal disease (estimated glomerular filtration rate (eGFR) <15 mL/min/1.73 m^2^) or long-term dialysis treatment; (3) patients who died within 24 h after PCI were also excluded because CIN could not be evaluated in these patients; (4) lack of data on preprocedural or postprocedural serum creatinine levels (SCr); (5) lack of data on preprocedural fibrinogen or albumin levels; and (6) intravascular administration of contrast medium within the last 7 or 3 days postoperatively. Finally, 565 eligible patients were selected. The study was approved by the ethics committee of the Fujian Provincial Hospital, China.

### 2.2. Study Protocol

PCI was performed according to standard clinical practice using standard guide catheters, guidewires, balloon catheters, and stents, via the femoral or radial approach. The contrast dose was determined at the discretion of the interventional cardiologist. All patients received nonionic, low-osmolarity contrast agents (either Iopamiron or Ultravist). In addition, all patients received normal saline at a rate of 1 ml/kg/h before the procedure and continued for 12 hours after the procedure (or 0.5 ml/kg/h for 12 hours in case of overt heart failure) according to the guidelines.

Patients were treated according to AHA/ACCF guidelines. Serum levels of albumin and fibrinogen were measured at the first or second day after admission. SCr was measured at admission and daily for 3 days after contrast administration. Biochemical parameters at admission were also measured, including serum glucose, uric acid, lipid profiles, hemoglobin, white blood cell and platelet counts, and glycated hemoglobin.

### 2.3. Definitions and Follow-Up

CIN was defined as either a 25% increase in baseline serum creatinine levels or a 0.5 mg/dL (44 *μ*mol/L) increase in absolute serum creatinine levels within 72 h after the contrast medium exposure [19]. Perihypotension was systolic blood pressure (SBP) <80 mmHg for at least 1 hour requiring inotropic support with medications or intraaortic balloon pump (IABP) within 24 hours periprocedurally. Follow-up data were obtained during an out-patient clinic visit or by phone. All patients included in the study were subject to follow-up for >1 year.

### 2.4. Statistical Analysis

We compared the baseline characteristics between the CIN group and non-CIN group. Normally distributed continuous variables were expressed as mean and standard deviation (SD) and analyzed using Student's *t*-tests. Nonnormally distributed variables were expressed as median and analyzed using Wilcoxon rank-sum test. Categorical variables were presented as percentages and analyzed using chi-square test or Fisher exact test. The receiver operating characteristic (ROC) curve analysis was used to determine the optimal cutoff value of FAR levels to detect CIN using the MedCalc statistical software (MedCalc Software, version 11.4.2.0). Univariate and multivariate logistic regression were performed to calculate odds ratios (OR) for risk factors of CIN. Variables that were found to be significant in the univariate analysis (*P* < 0.05) and a few variables that were confirmed to be significant in clinical practice were included in the multiple logistic regression analysis. Kaplan–Meier curve was used to assess the survival time between the group of FAR ≤0.106 and the group of FAR >0.106. A value of *P* < 0.05 was considered statistically significant. Statistical analyses were performed using SPSS version 20.0 (SPSS Inc, Chicago, Illinois, USA).

## 3. Results

A total of 565 patients were enrolled in this study. Baseline and procedural clinical characteristics are presented in Table 1, including the traditional risk factors for CIN between the patients with and without CIN. Of these, 29 (5.1%) developed CIN. Patients who developed CIN were older, as well as have lower lymphocyte values and higher serum creatinine, uric acid, and neutrophil values. They presented with more multivessel diseases and were more frequently treated with diuretics. Furthermore, these patients with CIN were more likely to have lower albumin and higher fibrinogen levels.

**Figure 1:**
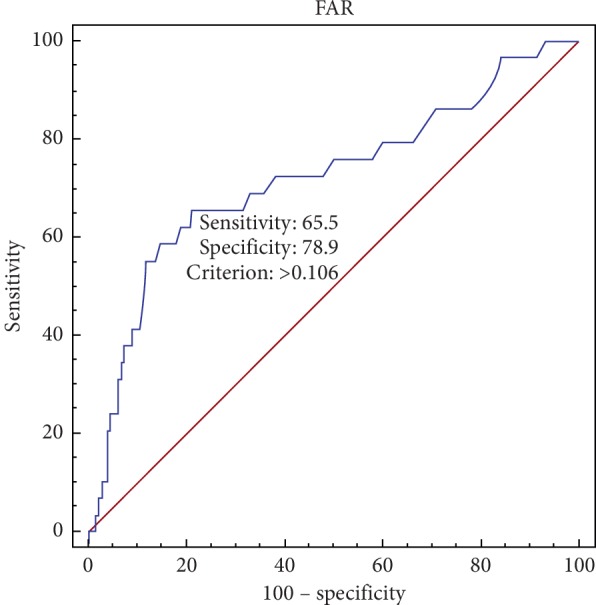
Receiver operator characteristic (ROC) curve analysis. ROC curve analysis demonstrated that an FAR cutoff value of 0.106 was optimal, exhibiting 65.5% sensitivity and 78.9% specificity for detecting contrast-induced nephropathy (CIN). The C-statistic was 0.721 (0.682–0.757).

ROC analysis indicated that a cutoff value of 0.106 for FAR could predict CIN with a sensitivity of 65.5% and a specificity of 78.9% (C statistic = 0.721; 95% CI, 0.682–0.757) (Figure 1). Univariate logistic regression determined that age >75 years, LVEF <45%, perihypotension, FAR >0.106, SCra >1.5 mg/dl, and neutrophil were associated with CIN (all *P* < 0.05). Multivariate analysis indicated that FAR levels (OR = 3.97; 95% CI, 1.61–9.80; *P*=0.003), peri‐hypotension (OR = 3.81; 95% CI, 1.49–9.72; *P*=0.005); age >75 years (OR = 4.54; 95% CI, 1.81–11.43; *P*=0.001) and LVEF <45% (OR = 3.70; 95% CI, 1.35–10.12; *P*=0.011) remained significant predictors for the development of CIN in patients after emergency PCI (Table 2).

**Figure 2:**
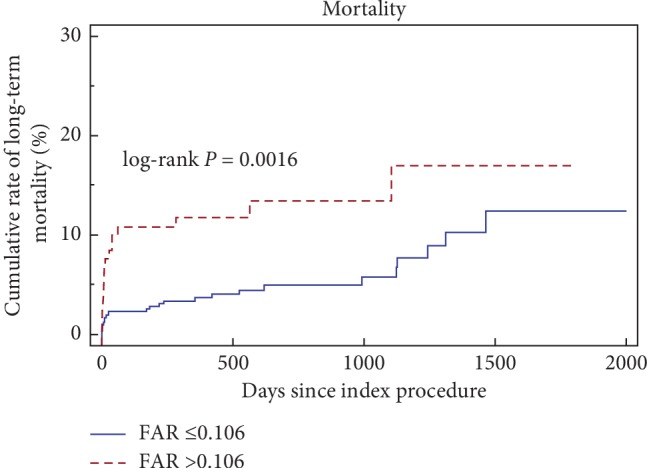
Kaplan–Meier curves demonstrate the cumulative mortality for patients based on the cutoff value of FAR levels (0.106).

Kaplan–Meier curve demonstrated that low levels of FAR presented high all-cause mortality based on the cutoff value of FAR (0.106) (*P*=0.0016, Figure 2).

## 4. Discussion

The main purpose of this study was set out to determine the diagnostic value of FAR in predicting CIN following emergency PCI in patients. We found that FAR was associated with an increased risk of developing CIN in patients after emergency PCI. After adjusting for confounders, the cutoff value of FAR for CIN prediction was 0.106 with a sensitivity of 65.5% and specificity of 78.9% for predicting CIN.

CIN is the one of the most common causes of hospital-acquired kidney injury due to the growing number of contrast-enhanced imaging studies, including PCI. It is known to raise morbidity and mortality and increase healthcare costs, as well as prolong hospitalization [20, 21]. The incidence of CIN was 3.3% in the interventional cardiology registry from Mayo Clinic including 7586 patients [22]. However, the incidence of CIN rises dramatically in patients with emergency PCI. The prevalence of CIN in our study in patients after emergency PCI was 5.1%, which is lower than studies ever reported before (19%) [23]. The discrepancy may be explained by 21 patients died within 24 h after PCI, who were at high risk for the development of CIN.

Systemic inflammatory indexes including NLR, PLR, and CRP have been introduced to as a significant independent marker for prediction of adverse outcome in oncologic disorders, cardiovascular diseases, and nephropathy. The study by Kocas et al. provided evidence that PLR was an independent predictor of CIN after angiography in patients with NSTE-ACS [10]. Sun et al. showed that in patients with STEMI undergoing primary PCI, both PLR and NLR were independent risk factors for the development of CIN [11]. Moreover, Yuan et al. demonstrated that NLR and CRP levels have high predictive values for CIN after an emergency PCI [14].

Accumulating studies have demonstrated that FAR, cooperating albumin and fibrinogen, is an important prognostic predictor in various cancer [16, 17]. Recently, Guclu et al. indicated that an elevated FAR was significantly correlated with ischemic retinal vein occlusion [24]. In the study by Karahan et al., FAR was significantly related to SYNTAX Score in predicting the severity of CAD in patients with STEMI [25]. Moreover, the study conducted by Ertas et al. evaluated the prognostic value of FAR to CIN in patients after carotid angiography. Multivariate logistic regression revealed FAR was independently predictive for CIN (OR = 1.029, 95% CI: 1.013–1.045, *P* < 0.001) [18]. Our study also revealed that FAR was a significant predictor of CIN in patients with emergency PCI.

Several studies have shown that albumin was significantly correlated with adverse outcomes in cardiovascular diseases. Recently, the predictive value of albumin in AKI was highlighted. A meta-analysis by Wiedermann et al. provided evidence that low levels of serum albumin were independent predictors of AKI and death following AKI [26]. Moreover, a prospective trial conducted by Lee et al. evaluated the effects of administration of 20% human albumin solution vs. saline on the incidence of postoperative AKI in adult patients with hypoalbuminemia, who were undergoing off-pump coronary artery bypass surgery. Multivariate logistic regression revealed a protective effect of albumin therapy on the renal function (OR 0.42; 95% CI 0.21–0.83; *P*=0.012) [27]. Murat et al. retrospectively assessed the impact of serum albumin levels on CIN occurrence in a cohort of 890 patients with ACS treated with PCI. The study demonstrated that serum albumin was inversely associated with AKI risk and independently predicted the occurrence of AKI along with other variables [28]. Our previous study also indicated that prealbumin, such as albumin, was independently associated with an increased risk of CI-AKI and long-term mortality in elderly patients undergoing elective PCI [29]. Although the exact mechanism underlying the association of low albumin levels with CIN was not still fully understood, there is a biological rationale for using albumin to predict the CIN in patients undergoing emergency PCI. One possible mechanism is that albumin plays a vital role in maintaining the oncotic pressure, increasing renal flow and urine output [30]. Albumin also possess antioxidant properties by scavenging the production of reactive oxygen species, preventing oxidative damage and delivering protective lysophosphatidic acid, which can increase the survival of cultured renal tubular cells [31, 32]. As negative acute-phase protein, low albumin levels are correlated with increased inflammatory status, and albumin possess anti-inflammatory effect by inhibiting the secretion of proinflammatory cytokines (TNF-*α*) and complement factors (C5a) through the modulation of the signaling systems between inflammatory cells [33, 34]. Furthermore, hypoalbuminemia may increase blood viscosity and disrupt endothelial function by increased concentrations of free lysophosphatidylcholine [35].

Fibrinogen is a serum glycoprotein and plays a critical role in the inflammatory process, such as the regulation of macrophage adhesion and the activation of cytokine/chemokine production. In addition, fibrinogen can stimulate interleukin-1*β* and tumor necrosis factor-*α* expression by macrophages and activate the macrophage adhesion, indicating the role of fibrinogen as an inflammatory marker [36]. Serum fibrinogen level is related to increased blood viscosity, which causes endothelial shear-stress damage [37]. Many previous studies have confirmed that there are strong associations between serum fibrinogen levels and increased risk for developing CIN [38], cardiovascular events [39], and stroke [40].

Our study had several limitations. First, all patients selected for this study were from a single center with a small population, and thus there was a selection bias during patient enrolment. Second, measurements of peak SCr levels might have been missed because of the variation in the measurement times, which may have caused an underestimation of the true incidence of CIN. Finally, whether the relationship between FAR and CIN is the effect of high levels of FAR or simply reflects the severity of the clinical status of these patients cannot be elucidated by this study. Despite these limitations, our results provide useful insights associating FAR and the incidence of CIN.

## 5. Conclusions

In conclusion, our study showed that elevated levels of preprocedural FAR were associated with the development of CIN in patients undergoing emergency PCI, and 0.106 was the optimal cutoff value of preprocedural FAR to predict CIN, which could guide the use of preventive measures and therapy to alleviate CIN.

## Figures and Tables

**Table 1:** Baseline clinical features in patients with and without CIN.

	CIN (−) (*n* = 536)	CIN (+) (*n* = 29)	*P* value
Demographics			
Age, years	62.80 ± 12.08	72.90 ± 11.33	<0.001
Age >75 years, *n* (%)	83 (15.5%)	14 (48.3%)	<0.001
Sex, female, *n* (%)	70 (13.1%)	1 (3.4%)	0.158
Medical history			
Smoker	318 (59.3%)	21 (72.4%)	0.161
Prior PCI, *n* (%)	17 (3.2%)	1 (3.4%)	1.000
Hypertension, *n* (%)	309 (57.6%)	17 (58.6%)	0.918
Diabetes, *n* (%)	138 (25.7%)	11 (37.9%)	0.147
Laboratory measurements			
Albumin, g/L	39.79 ± 3.95	37.14 ± 5.21	0.012
Creatinine, mg/dl	0.85 ± 0.26	1.14 ± 0.61	0.017
SCr >1.5 mg/dl, *n* (%)	14 (2.6%)	5 (17.2%)	0.002
WBC, 10^9^/l	12.10 ± 3.86	13.54 ± 4.49	0.053
Hemoglobin, g/l	140.86 ± 15.72	131.24 ± 26.17	0.06
Hematocrit	0.56 ± 2.48	1.75 ± 7.32	0.392
Neutrophil, 10^9^/l	9.83 ± 3.77	11.50 ± 4.06	0.021
Cholesterol, mmol/l	4.85 ± 1.14	4.44 ± 1.12	0.062
Triglyceride, mmol/l	1.54 ± 1.17	1.12 ± 0.44	0.057
Low-density lipoprotein, mmol/l	3.28 ± 1.01	2.92 ± 1.07	0.059
Fibrinogen, g/l	3.51 ± 0.94	4.14 ± 0.96	<0.001
LVEF <45%, *n* (%)	37 (6.9%)	10 (34.5%)	<0.001
Procedure performed			
Perioperative hypotension, *n* (%)	183 (34.1%)	20 (69.0%)	<0.001
Contrast volume >200 ml, *n* (%)	376 (70.1%)	23 (79.3%)	0.291
IABP	10 (1.9%)	4 (13.8%)	0.004

Data are presented as the means ± standard deviations or as numbers and percentages. CIN—contrast-induced nephropathy; PCI—percutaneous coronary intervention; SCr—serum creatinine; WBC—white blood cell; LVEF—left ventricular ejection fraction; IABP—intraaortic balloon pump.

**Table 2:** Univariate and multivariate logistic regression analyses for CIN.

Risk factors	Univariate logistic regression			Multivariate logistic regression		
	OR	95% CI	*P* value	OR	95% CI	*P* value
Age >75 years	5.09	2.37–10.95	<0.001	4.54	1.81–11.43	0.001
FAR	7.11	3.22–15.72	<0.001	3.97	1.61–9.80	0.003
SCr >1.5 mg/dl	7.77	2.59–23.00	<0.001	2.42	0.61–9.60	0.209
LVEF <45%	7.10	3.08–16.37	<0.001	3.70	1.35–10.12	0.011
Perihypotension	4.29	1.91–9.60	<0.001	3.81	1.49–9.72	0.005
Neutrophil	1.11	1.02–1.21	0.022	1.05	0.94–1.18	0.380

CIN—contrast-induced nephropathy; SCr—serum creatinine; OR—odds ratio; CI—confidence interval; FAR—fibrinogen-to-albumin ratio; LVEF—left ventricular ejection fraction.

## Data Availability

The patient data used to support the findings of this study are restricted by the Fujian Provincial Hospital Clinical Research Ethics Committee in order to protect patient privacy. Data are available from the corresponding author (lky7411@sina.com) for researchers who meet the criteria for access to confidential data
